# The Effect of Nurse Navigators in Digital Remote Monitoring in Cancer Care: Case Study Using Structural Equation Modeling

**DOI:** 10.2196/66275

**Published:** 2025-03-28

**Authors:** Etienne Minvielle, Joel Perez-Torrents, Israa Salma, Philippe Aegerter, Marie Ferrua, Charles Ferté, Henri Leleu, Delphine Mathivon, Claude Sicotte, Mario Di Palma, Florian Scotté

**Affiliations:** 1 Interdisciplinary Department for the Organization of Patient Pathways Gustave Roussy Villejuif France; 2 i3-CRG, École Polytechnique Institut Polytechnique de Paris Palaiseau France; 3 Université de Versailles Saint-Quentin-en-Yvelines Versailles France; 4 Centre Hospitalier d'Argenteuil Argenteuil France; 5 Resilience Care Paris France; 6 Public Health Expertise Paris France; 7 Healthcare Management Department Université de Montreal Montreal, QC Canada

**Keywords:** digital remote monitoring, nurse navigators, patient care, oncology, toxicity, patient satisfaction, hospitalization

## Abstract

**Background:**

The purpose of digital remote monitoring (DRM) is improving cancer care management. However, its effectiveness largely depends on the role of nurse navigators (NNs) within these systems to process data and lead action.

**Objective:**

This study aims to fill gaps in our understanding of the role of NNs within a specific system, drawing on the Cancérologie parcours région Ile-de-France (CAPRI) DRM program applied to oncology patients.

**Methods:**

The CAPRI DRM, targeting patients taking oral anticancer agents, combines digital interfaces with NN interventions. A phase 3 randomized controlled trial involving 559 patients assessed its safety and efficacy, with the primary end point being the relative dose intensity. This report focuses on patients in the CAPRI arm, evaluating the impact of NN interventions on outcomes such as toxicity, hospitalization, and emergency visits. Data on patient characteristics, NN interventions, and patient satisfaction surveys were analyzed using structural equation modeling.

**Results:**

The study included 187 patients. Patient characteristics were significantly correlated with outcomes. Across all the models we used, the quality of NN interventions was consistently associated with higher patient satisfaction, with correlation coefficients ranging from 0.332 (95% CI 0.154-0.510; *P*<.001) to 0.366 (95% CI 0.182-0.550; *P*<.001). The number of grade ≥3 toxicity events correlated positively with NN referrals to oncologists. Hospitalization length was positively related to NN referral (coefficient 0.102, 95% CI 0.051-0.153; *P*<.001) and inversely to NN advice (coefficient –0.045, 95% CI –0.096 to 0.006; *P*=.08). Emergency visits showed a negative correlation with NN actions (coefficient –0.478, 95% CI –0.923 to 0.033; *P*=.04) and a positive correlation with NN calls and referrals (coefficient 0.516, 95% CI 0.069-0.963; *P*=.02).

**Conclusions:**

This study shows the central role of NNs in making DRM effective. Despite the study’s limitations, these results support the design of DRM as a hybrid model of automated digital tools and human support. Future research should explore the applicability of such a DRM model in various clinical settings to clarify the optimal association between automated systems and NN expertise.

**Trial Registration:**

ClinicalTrials.gov NCT02828462; https://www.clinicaltrials.gov/study/NCT02828462

## Introduction

### Background

The tremendous uptake of digital care (mobile apps and web portals) has paved the way for improving the use of remote patient monitoring (ie, the digitally remote collection of patient-generated data that is automatically sent to health care providers), and it has created opportunities to further integrate digital remote monitoring (DRM) into the delivery of various health services. Several clinical trials implementing DRM tools showed that their use may improve the quality of care compared to standard care, reduce costs, supplement (or replace) in-hospital care, and improve management of clinical events in different chronic conditions [1,2], including cancer [3-7].

However, implementing supportive digital remote monitoring interventions remains challenging, with concerns about potential overuse and exacerbation of health inequalities due to the digital divide [8-11]. Successful DRM implementation relies on several factors, combining patient-reported outcomes, carefully designed technology, and specialist health care professionals’ expertise [7-12].

### What Role Is Played by Nurse Navigators?

Considering the importance of professional expertise, the actions of nurse navigators (NNs) may become crucial. Their expertise lies in clinical care and patient navigation, meaning that NNs can enhance the usability and safety of DRM technologies [12]. They can be a critical link between patients and the health care system, ensuring digital tools are effectively integrated into patient care. NNs can interpret data from digital tools, provide timely interventions, and guide patients through their treatment journey [12-14].

Despite the postulated importance of NNs in DRM, more research is needed to explore the extent to which they contribute to the effectiveness of these systems, especially in oncology. This study aims to fill this gap by examining the impact of NNs in the Cancérologie parcours région Ile-de-France (CAPRI) DRM program, considering factors such as toxicity management and care use.

## Methods

### An Example of DRM: CAPRI

CAPRI is an application of DRM in oncology. It specifically targets patients receiving oral anticancer agents, focusing on improving adherence, managing toxicities, and enhancing overall patient care. The core of CAPRI lies in its dual components: a digital interface (including a smartphone app and a web portal) and the active involvement of NNs in patient monitoring and intervention [14]. NNs involved in the CAPRI study received in-house training during two 2-hour sessions on how to use the application.

The safety and efficacy of CAPRI were assessed in a phase 3 randomized controlled trial (RCT) comparing the CAPRI DRM intervention against the standard of care. The trial included 559 randomized patients followed for 6 months. The study’s primary end point was the relative dose intensity (RDI), defined as the ratio of the dose delivered over time to the prescribed dose intensity. Secondary end points included treatment adherence, toxicity management, patient-reported outcomes, and overall quality of life. The trial’s design and results have been published elsewhere [15]. The main results showed that the CAPRI group (n=272) showed a significant improvement in RDI (93.4% vs 89.4%; *P*=.04), an enhanced patient experience (Patient Assessment of Chronic Illness Care score 2.94 vs 2.67; *P*=.01), reduced hospitalization length (2.82 vs 4.44 days; *P*=.02), and decreased grade ≥3 toxicities (27.6% vs 36.9%; *P*=.02).

This study is an ancillary analysis of the 272 patients included in the CAPRI intervention arm. Its objective is to detail the mechanisms of the NN interventions’ effects on patient outcomes in the intervention arm.

### Data Collection

**Figure 1:**
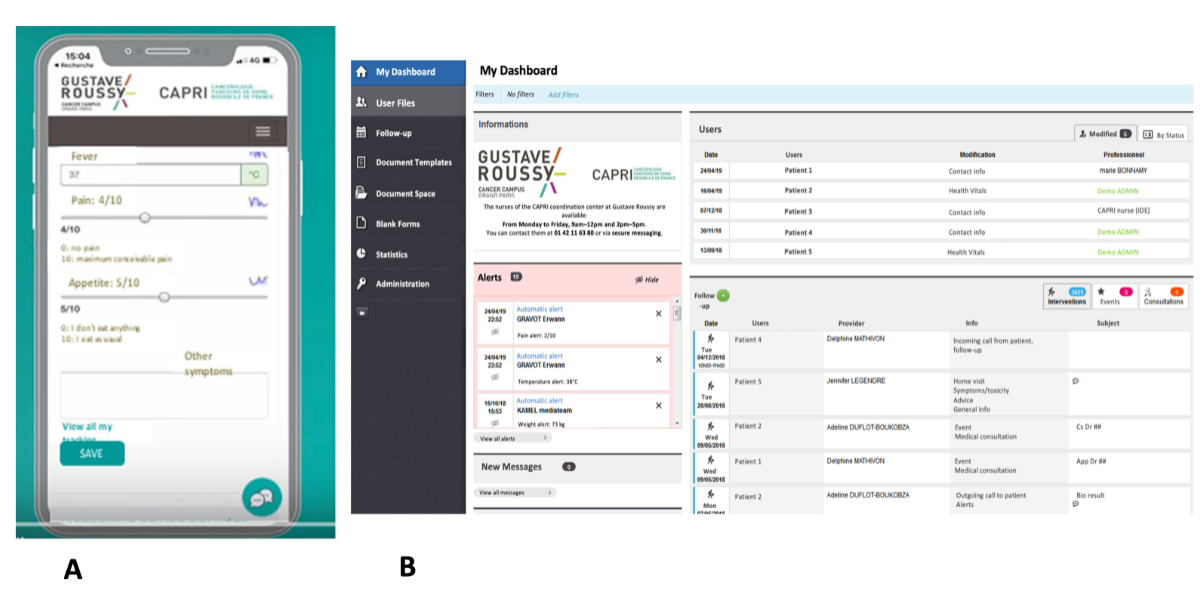
Screenshots of the patient smartphone app (A) and nurse navigators’ dashboard (B).

Three distinct types of data collected during the RCT were used: patient characteristics and outcomes, including demographics and outcomes that have been previously published [15]; NN interventions, extracted from the NN dashboard used during the trial (Figure 1); and patient perceptions and satisfaction, based on a dedicated patient questionnaire administered at the end of the RCT to assess patients’ use, perceived utility, and satisfaction with the program. Data were available for 69% of patients (n=187).

### Data Analysis

**Figure 2:**
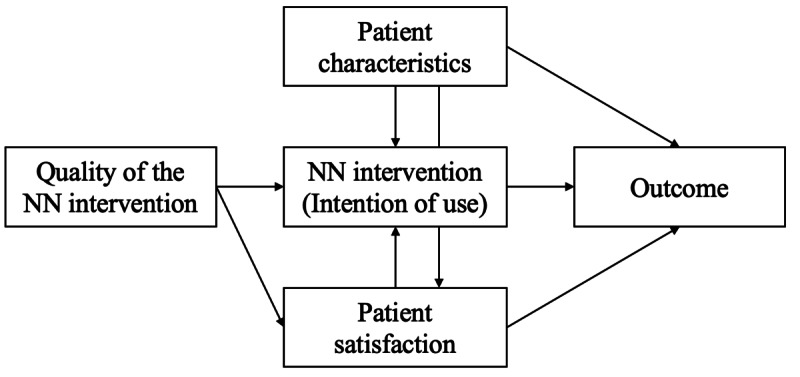
Structural modeling framework used to explore the effects of nurse navigator (NN) interventions on outcomes.

The role of NN interventions in outcomes, including toxicity, hospitalization length, and emergency service visits, was explored using a structural equation modeling approach. As shown in Figure 2, the framework used draws inspiration from the DeLone and McLean [16] model, which explains the success of information systems like DRM based on the roles of system quality, user satisfaction, and use.

The structure used considers that differences in outcomes are mediated through the NN interventions after adjusting to individual patient’s characteristics. The quality of the NN interventions and patients’ satisfaction were assessed using the patient questionnaire. Through factor analysis, it was verified that the associated questionnaire items corresponded to shared underlying dimensions, thereby allowing the reduction of these items to the unified latent variables used in the structural model.

CAPRI use was based on the number and frequency of NN interventions throughout the RCT, separated into (1) the number of inbound or outbound calls, (2) actions taken (appointment management, electronic health record management, information about administrative issues), (3) referrals (external health care providers, internal oncologists), (4) advice to patients, and (5) coordination actions. Patient characteristics included sociodemographic data, fitness (World Health Organization score) and metastatic cancer status. Before introduction into the model, variables were standardized to account for different measurement scales, while missing data were imputed using a complete information maximum likelihood approach.

Final models were selected using an iterative process based on the model’s fit, which was evaluated using several global indicators, including the standardized root mean square residual, adjusted goodness of fit, Tucker-Lewis index, comparative fit index, and root mean squared error of approximation. Our sample size (n=187) refers to general rules adopted for structural equation models with a small numbers of variables (4 to 7, depending on the model) [17]. A complete model and methodological description are available in Multimedia Appendix 1.

### Ethical Considerations

This study was a secondary analysis based on the results of the CAPRI study, which was conducted according to applicable laws and regulations and the Declaration of Helsinki and was approved by an ethics committee (CPP Paris-Ile-de-France IV [2016/20SC] and US Department of Health and Human Services (00003835). The study was registered on ClinicalTrials.gov (NCT02828462). All patients included in the study provided informed consent to participate in the study. The informed consent form clarified that the collected data were part of the CAPRI study and would be used for primary and secondary analyses. To protect participants’ privacy, all data were anonymized. No compensation was offered to participants.

## Results

### Patient Characteristics

Overall, 187 patients from the intervention arm of the CAPRI trial were included. The characteristics of the respondents were similar to those of the complete set of patients, except for a longer follow-up period, as shown in Table 1.

**Table 1:** Patient characteristics.

Characteristics		Participants in this study (n=187)		Participants in the CAPRI^a^ study intervention arm (n=272)		*P* value
Age (years), mean (SD)		59.8 (14.2)		59.1 (14.0)		.59
Female participants, n (%)		103 (55.8)		156 (57.3)		.63
**Performance status^b^, n (%)**						.24
	WHO^c^ 0	92 (49.2)	121 (44.5)			
	WHO 1	82 (43.9)	120 (44.1)			
Metastatic cancer, n (%)		147 (78.6)		222 (81.6)		.42
Follow-up duration (weeks), mean (SD)		148.7 (48.3)		133.0 (54.2)		.02

^a^CAPRI: Cancérologie parcours région Ile-de-France.

^b^Performance status reflects cancer patients’ functional ability, ranging from normal activity (0) to completely bedridden (4).

^c^World Health Organization.

As expected, patients’ characteristics, including age, performance status, and metastatic stage, were significantly associated with the selected outcomes (Multimedia Appendix 1, Figures S3 to S5).

### The Role of NN Interventions

**Figure 3:**
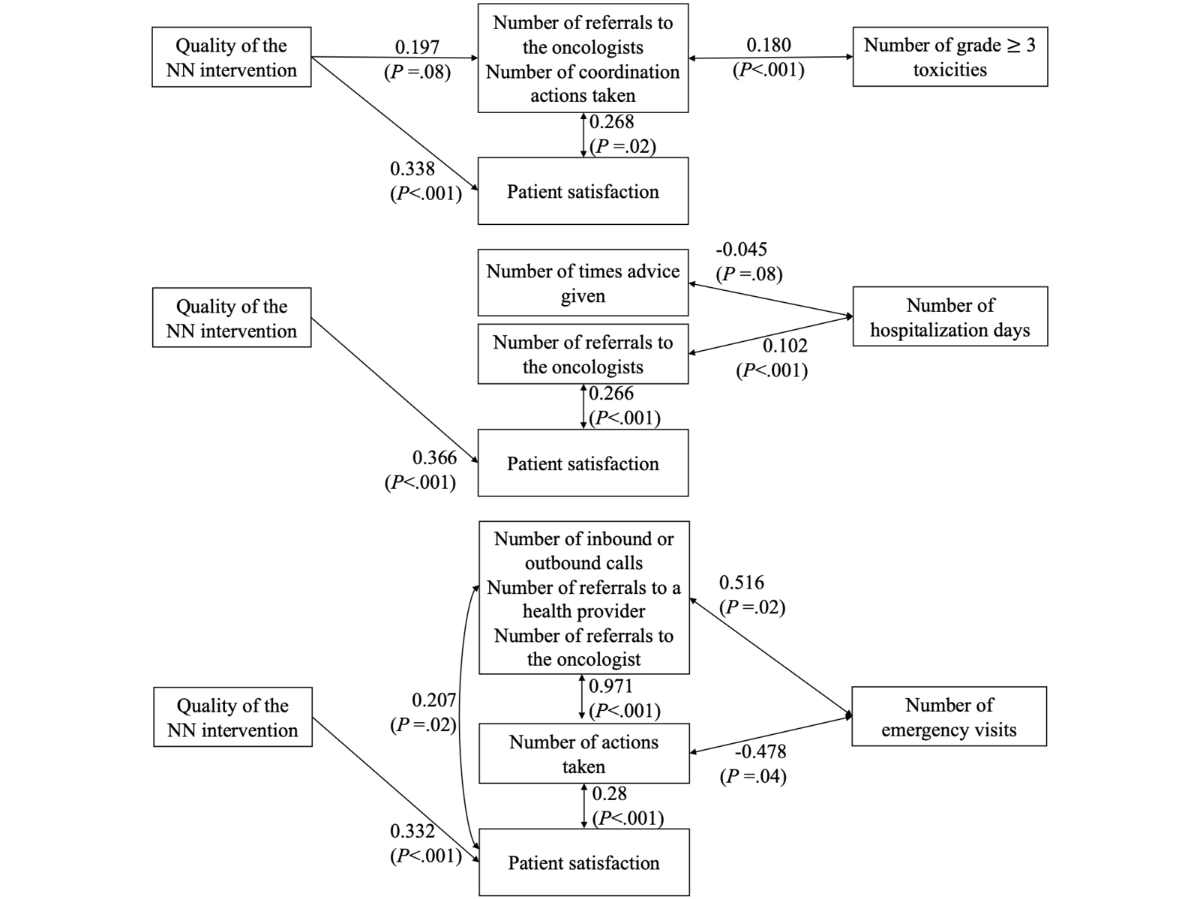
Simplified results (correlation coefficients) of the final structural models (excluding patient characteristics) for each outcome. NN: nurse navigator.

Structural model results, which do not show patients’ characteristics, are presented in Figure 3.

Across all the models we used, the quality of NN interventions was consistently associated with higher patient satisfaction, with correlation coefficients ranging from 0.332 (95% CI 0.154-0.510; SE 0.091; *z*=3.64; *P*<.001) to 0.366 (95% CI 0.182-0.550; SE 0.094; *z*=3.90; *P*<.001). Likewise, patient satisfaction was consistently and positively associated with NN interventions, with similarly strong correlation coefficients ranging from 0.207 (SE 0.088, 95% CI 0.035-0.380; *z*=2.341; *P*=.02) to 0.283 (SE 0.079, 95% CI 0.128-0.438; z=3.598; *P*<.001).

The number of grade ≥ 3 toxicity events was positively associated with the number of referrals to oncologists and coordination actions. Hospitalization length was positively correlated with the number of referrals to oncologists (coefficient 0.102, 95% CI 0.051-0.153; SE 0.026; *z*=3.90; *P*<.001) and negatively associated with the number of times advice was given (coefficient –0.045, 95% CI –0.096 to 0.006; SE 0.026; *z*=–1.73; *P*=.08). Emergency visits were negatively related to the number of actions taken (coefficient –0.478, 95% CI –0.923 to –0.033; SE 0.227; *z*=–2.10; *P*=.04) and positively associated with the number of calls and the number of referrals (coefficient 0.516, 95% CI 0.069-0.963; SE 0.228; *z*=2.26; *P*=.02).

## Discussion

This study corroborates past findings that nurse-led interventions in a DRM program for cancer patients can impact health outcomes [9-11]. The actions taken by NNs within CAPRI were associated with reduced emergency service use and hospitalization length. This suggests that NNs are critical in providing appropriate advice and avoiding unnecessary emergency service use and hospital interventions. The positive associations between emergency service use, hospitalization length, and grade ≥ 3 toxicity events and NN interventions, such as coordination or referrals, likely reflect a prioritization in monitoring and treating severe cases. This also highlights the versatility of NNs in managing most patient interaction and orientation without direct oncologist referrals, confirming their potential in delegating certain oncologist tasks [18].

These findings emphasize the importance of considering DRM as a hybrid model that combines digital tools with human support, especially in complex cases. Additionally, patients’ preferences for different platforms (phone or web based) or reluctance to use digital platforms highlights the need for flexibility and human availability in DRM implementation [19].

Despite its contributions, this study had limitations, including its single-center nature, which might affect the external validity of the findings. This hybrid model’s application to other cancer conditions and its generalizability requires further investigation.

In conclusion, the study illuminates the role of skilled nurses in DRM implementation, suggesting the need for future research to explore the division of tasks between automated alert systems and human expertise, particularly in managing complex cases and cancer pathways. The potential managerial implications underline the importance of clearly defining the role of NNs and considering their activity in payment models for digital remote monitoring systems [20].

## Appendix

Multimedia Appendix 1 Supplementary material.

Multimedia Appendix 2 Checklist of iCHECK-DH guidelines.

## Abbreviations

CAPRI: Cancérologie parcours région Ile-de-France
DRM: digital remote monitoring
iCHECK-DH: Guidelines and Checklist for the Reporting on Digital Health Implementations
NN: nurse navigator
RCT: randomized controlled trial
RDI: relative dose intensity

## Data Availability

All data generated or analyzed during this study are included in this published article and its supplementary information files.
