# Supplementary material for: The Effect of Nurse Navigators in Digital Remote Monitoring in Cancer Care: Case Study Using Structural Equation Modeling
Source: J Med Internet Res. 2025 Mar 28;27:e66275. doi: 10.2196/66275 (PMC11992497; doi:10.2196/66275)
Supplement: Multimedia Appendix 1 [file jmir_v27i1e66275_app1.docx]

**Supplementary Material**

***Multimedia Appendices***

**Methodology**

***Data source***

This study is based on data collected during the CAPRI trial.

The CAPRI study, a Phase 3 randomized clinical trial (RCT) (NCT02828462), explored the efficacy of a nurse navigator (NN)-led program in managing treatment for metastatic cancer patients. Participants, all on approved oral anticancer medications, were divided equally into two groups: one received standard care plus a combined nurse navigator follow-up and digital platform support (web portal and smartphone app). At the same time, the other underwent conventional symptom monitoring by their oncologist over six months. The main goal was to optimize treatment dosing, with secondary aims focusing on severe toxicities, patient experiences, hospital stay frequency and length, treatment response, survival rates, and quality of life. Out of 559 patients, those in the experimental group (n=272) showed a higher relative dose intensity (93.4% vs. 89.4%, P = 0.04), an enhanced patient experience (Patient Assessment of Chronic Illness Care score 2.94 vs. 2.67, P = 0.01), fewer hospitalization days (2.82 vs. 4.44 days, P = 0.02), and a reduction in treatment-related severe toxicities (27.6% vs 36.9%, P = 0.02).^1^

***Data collected***

This study is based on an ancillary analysis of data collected for patients in the intervention arm. These include:

- Demographic, socioeconomic, and clinical variables include performance status (WHO score), comorbidities, primary tumor site, primary tumor stage, and prior treatment lines.
- **Relative Dose Intensity (RDI)**, defined as the ratio of the dose delivered over time to the initially prescribed dose (primary endpoint).
- Patient Adherence to Oral Anticancer Therapy, measured using a dedicated questionnaire and/or the Medication Event Monitoring System.
- Quality of Life, assessed using the EORTC QLQ-C30 questionnaire.
- Patient Experience, measured with the PACIC score.
- Tumor Response (RECIST 1.1), progression-free survival and overall survival, evaluated by investigators.
- Grade ≥3 Toxicities, graded according to the NCI-CTCAE v4.03 classification.
- Resource Data collected monthly, including hospitalizations, emergency visits and support care.
- Process evaluation includes web portal data use and NN intervention. NN intervention data, including the timing, frequency and number of NN interventions for each patient.
- Patients perceived utility and satisfaction with the program collected at the end of the trial based on a 69-item questionnaire covering phone/computer equipment and web, ease of use and perceived utility of the web platform, ease of interaction and perceived utility of the NN intervention and overall satisfaction of the program.

***Endpoints***

The impact of the NN intervention was assessed on the following endpoints:

- Number of grade ≥3 Toxicities reported during the study period (by quintile)
- Number of hospitalization days during the study period (< or ≥ median)
- Number of emergency visits during the study period (< or ≥ median)

***Predictor variables***

The following predictor variables were considered in the analysis:

- Demographic (age, sex), socioeconomic, and clinical variables (performance status, comorbidities (yes/no), primary tumor stage (metastasis, local or locally advanced)
- NN intervention intensity is separated into the number of interventions made by the NN (inbound or outbound call), the number of actions taken by the NN (appointment management, electronic health record management, information about administrative issues), the number of referrals by the NN to a health provider, the number of referrals to oncologists, the number of advice given to the patients and the number of coordination action taken (orientation to a hospital, or an ambulatory care’s organization).
- The quality of the NN intervention (perceived utility and ease of interaction) and overall satisfaction with the program collected at the end of the trial based on the dimensions of the 69-item questionnaire. Items with a response rate below 50% were excluded from the analysis.

***Data management***

Variables were standardized to avoid the influence of different scales of measurement when comparing coefficients of predictors. Missing answers were credited with a complete information maximum likelihood approach (FIML), a case-wise maximum likelihood estimation function with the Lavaan package in R^2^.

To validate that the selected questionnaire items accurately represent the constructs of “quality of the Nurse Navigator (NN) interventions” and “patient satisfaction,” series of exploratory factor analysis (EFA) were performed using the factorial function from the Lavaan package in R. This statistical method identifies underlying relationships between measured variables and groups them into factors based on their correlations.

Once confirmed that the selected questionnaire items represented a common dimension, the factor analysis was used to consolidate the items for the quality of the NN interventions and overall satisfaction into two singular, comprehensive variables. Items 45, 46 and 47 of the questionnaires were combined into a single construct for the quality of the NN' interventions, and items 53, 54, 59, 60, 61, 63, 64, 65, 67 and 68 were combined into a single construct for patient satisfaction. These items are evaluated from 1 (Don’t agree at all) to 4 (Totally agree).

The items included in each construct are consistent with the theoretical definitions of “quality of NN interventions” and “patient satisfaction.” Likewise, the factor analysis supports the grouping of items based on their loadings on the respective factors. (Details on the EFA results are provided below.)

Item 45. I needed help contacting the NN.

Item 46. The NN are at my disposal.

Item 47. NN are available.

Item 53. Being able to communicate with the NN is reassuring.

Item 54. The fact that the NN can be in contact with the health professionals involved in my care is reassuring.

Item 59. The usefulness of CAPRI monitoring.

Item 60. CAPRI monitoring helps with treatment monitoring.

Item 61. CAPRI monitoring helps to manage side effects.

Item 63. The usefulness of CAPRI monitoring for those around you.

Item 64. CAPRI monitoring is reassuring for those around me.

Item 65. I would recommend CAPRI monitoring, which benefits me.

Item 67. CAPRI monitoring helped me get involved in my care.

Item 68. CAPRI monitoring meets my expectations and needs regarding my care.

***Structural Model Selection***

Structural models were fitted for each endpoint, employing an iterative approach to identify the most suitable model based on goodness-of-fit criteria. Variables were retained in the final model if their p-values exceeded the .1 threshold. The following measures were used for goodness-of-fit, with their respective threshold requirement:

- standardized root mean square residual (SRMR) to show the model fit the data sufficiently (a value < 0.08 was accepted)
- adjusted goodness of fit (AGFI) to check parsimony (a value > 0.9 was accepted)
- Tucker-Lewis index (TLI) and comparative fit index (CFI) as the sample was small (for both, a value > 0.95 was accepted)
- Root mean squared error of approximation (RMSEA) a parsimony-adjusted index (a value < 0.06 was accepted).

Final models were selected based on whether they met the fit requirements.

**Detailed Results**

***Exploratory Factor Analysis***

The exploratory factor analysis (EFA) results for all dimensions are detailed hereafter.

**Supplementary Table 2**. Factor loadings and cumulative variance explained

|  | Factor 1 | Factor 2 | Factor 3 | Factor 4 | Factor 5 | Factor 6 | Factor 7 | Factor 8 |
| --- | --- | --- | --- | --- | --- | --- | --- | --- |
| SS loadings | 5.407 | 5.227 | 3.563 | 2.951 | 2.674 | 2.325 | 1.368 | 1.116 |
| Proportion Var | 0.118 | 0.114 | 0.077 | 0.064 | 0.058 | 0.051 | 0.03 | 0.024 |
| Cumulative Var | 0.118 | 0.231 | 0.309 | 0.373 | 0.431 | 0.481 | 0.511 | 0.535 |

Test of the hypothesis that 8 factors are sufficient.

The chi-square statistic is 905.49 on 695 degrees of freedom.

The p-value is 1.13e-07

The results of the EFA suggested a clear factor structure, with a distinct set of items loading highly on each factor. These loadings, all above the commonly accepted threshold of 0.5, indicated a strong association of items with their respective factors, suggesting that each factor represented a coherent construct. The cumulative variance explained by the extracted factors was substantial, indicating that these factors captured a significant portion of the information contained in the original items. The factor analysis provided robust evidence for the validity of the factor structure of the questionnaire, with the identified factors demonstrating strong internal consistency and construct validity.

Factor 4 was associated with items representing the perceived utility of NN interventions (items 45, 46, and 47 of the questionnaire). Both items 46 and 47 loaded very highly on Factor 4, indicating they measure the same underlying construct related to the availability and accessibility of NN. Item 45 had low communalities, however, based on theoretical importance, it may still be considered in the construct.

**Supplementary Table 3.** Factor loadings for the perceived utility of NN interventions

|  | Factor 1 | Factor 2 | Factor 3 | Factor 4 | Factor 5 | Factor 6 | Factor 7 | Factor 8 |
| --- | --- | --- | --- | --- | --- | --- | --- | --- |
| Item 45 |  | 0.101 |  | 0.155 | 0.175 |  |  |  |
| Item 46 |  | 0.100 |  | 0.980 |  | 0.128 |  |  |
| Item 47 |  | 0.100 |  | 0.964 |  | 0.119 |  |  |

Factors 3 and 6 were associated with items representing satisfaction with the program for NN (items 53 and 54 on factor 6) and overall intervention respectively (items 59, 60, 61, 63, 64, 65, 67 and 68 on factor 3).

**Supplementary Table 4**. Factor loadings for patient satisfaction

|  | Factor1 | Factor2 | Factor3 | Factor4 | Factor5 | Factor6 | Factor7 | Factor8 |
| --- | --- | --- | --- | --- | --- | --- | --- | --- |
| Item 53 |  |  | 0.173 | 0.116 | 0.189 | 0.931 |  | -0.159 |
| Item 54 |  |  | 0.174 |  | 0.152 | 0.956 |  | 0.11 |
| Item 59 | 0.19 | 0.139 | 0.583 |  | 0.185 | 0.168 | 0.123 |  |
| Item 60 | 0.191 | 0.229 | 0.619 |  |  |  |  |  |
| Item 61 | 0.313 | 0.124 | 0.562 | 0.149 |  |  |  |  |
| Item 63 | -0.194 | 0.122 | 0.487 | 0.184 | -0.135 | 0.158 |  | -0.187 |
| Item 64 |  | 0.179 | 0.49 |  |  |  |  | -0.289 |
| Item 65 | 0.136 |  | 0.592 |  |  | 0.16 |  | -0.196 |
| Item 67 |  | 0.13 | 0.679 | 0.143 |  |  |  | 0.135 |
| Item 68 |  |  | 0.681 | -0.136 |  |  |  | 0.144 |

***Structural Model Selected***

The following table shows the final model fit values.

**Supplementary Table 5**. Final Model Fit

|  | Thresholds | Number of hospitalization days | Number of emergency visits | Support care | Number of grades≥3 Toxicities |
| --- | --- | --- | --- | --- | --- |
| Chi-square |  | 192.367 (df = 128, p-value = 0.000) | 59.897 (df = 58,  p-value = 0.407) | 310.880 (df = 181,  p-value = 0.00) | 55.761 (df = 50, p-value = 0.267) |
| SRMR | < 0.08 | 0.074 | 0.062 | 0.063 | 0.042 |
| AGAFI | > 0.9 | 0.985 | 0.993 | 0.972 | 0.991 |
| TLI | > 0.95 | 0.948 | 0.999 | 0.950 | 0.992 |
| CFI | > 0.95 | 0.956 | 0.999 | 0.957 | 0.994 |
| RMSEA | < 0,06 | 0.043 (p-value = 0.821) | 0.011(p-value = 0.995) | 0.051 (p-value = 0. 397) | 0.021 (p-value= 0.978) |

Full final model descriptions for each endpoint are shown below.

**Supplementary Figure 3**. Full structural model for the number of grade ≥3 Toxicities (* p < 0.1, ** p < 0.05, *** p < 0.01)

**Supplementary Figure 4**. Full structural model for the number of hospitalization days (* p < 0.1, ** p < 0.05, *** p < 0.01)

**Supplementary Figure 5**. Full structural model for the number of emergency visits (* p < 0.1, ** p < 0.05, *** p < 0.01)

**References**

1. Mir O, Ferrua M, Fourcade A, et al. Digital remote monitoring plus usual care versus usual care in patients treated with oral anticancer agents: the randomized phase 3 CAPRI trial. Nat Med. Jun 2022;28(6):1224-1231. [doi:10.1038/s41591-022-01788-1]

2. Gana K, Broc G. Structural equation modeling with lavaan. John Wiley & Sons; 2019.
